# Influence of orthodontic treatment on temporomandibular disorders. A systematic review

**DOI:** 10.4317/jced.52037

**Published:** 2015-04-01

**Authors:** Felipe J. Fernández-González, Aránzazu Cañigral, José L. López-Caballo, Aritza Brizuela, Isabel Moreno-Hay, Jaime del Río-Highsmith, José A. Vega

**Affiliations:** 1 Departament of Surgery and medical-surgical specialties. University of Oviedo, Spain; 2 Department of Oral Implantology, School of Medicine and Dentistry, University of the Basque Country, Spain; 3 Department of Orofacial Prosthetics of the Complutense University of Madrid, Spain; 4 Department of Stomatology I. Faculty of Dentsitry. Complutense University of Madrid, Spain; 5 Departament of Morphology and Cell Biology. University of Oviedo, Spain; 6 Facultad de Ciencias de la Salud. Universidad Autónoma de Chile, Chile

## Abstract

**Objectives:**

The aim of this literature systematic review was to evaluate the possible association between malocclusions, orthodontic treatment and development of temporomandibular disorders. Material and Methods: A search was carried out on PubMed-Medline database from January 2000 to August 2013 using the keywords “orthodontics and temporomandibular disorders”, “orthodontics and facial pain” and “malocclusion and temporomandibular disorders”. Human studies included in the study were those assessing signs and symptoms of temporomandibular disorders in relation to orthodontic treatment.

**Material and Methods:**

A search was carried out on PubMed-Medline database from January 2000 to August 2013 using the keywords “orthodontics and temporomandibular disorders”, “orthodontics and facial pain” and “malocclusion and temporomandibular disorders”. Human studies included in the study were those assessing signs and symptoms of temporomandibular disorders in relation to orthodontic treatment.

**Results:**

The search strategy resulted in 61 articles. After selection according to the inclusion/exclusion criteria 9 articles qualified for the final analysis. The articles which linked orthodontics and development of temporomandibular disorders showed very discrepant results. Some indicated that orthodontic treatment could improve signs and symptoms of temporomandibular disorders, but none of them obtained statistically significant differences.

**Conclusions:**

According to the authors examined, there is no evidence for a cause-effect relationship between orthodontic treatment and temporomandibular disorders, or that such treatment might improve or prevent them. More longitudinal studies are needed to verify any possible interrelationship.

** Key words:**Malocclusion and temporomandibular disorders, orthodontics and facial pain, orthodontics and temporomandibular disorders, temporomandibular disorders, temporomandibular dysfunction.

## Introduction

The American Association of Dental Research (AADR) recognizes that temporomandibular disorders (TMD) encompass a group of musculoskeletal and neuromuscular conditions that involve the temporomandibular joints, the masticatory muscles, and all associated tissues. They also are frequently associated with acute or persistent pain, and the patients often suffer some other painful disorders (comorbidities). In the chronic forms of TMD, pain may cause work absenteeism or some degree of impairment, resulting in an overall reduction in the quality of life (1).

TMD are considered multifactorial etiology conditions involving trauma, anatomical, pathophysiological, and psychosocial factors (2,3). The role of morphological and functional occlusion in their development has been matter of debate for a long time. Occlusal interferences, class II or III malocclusions, anterior open bite, excessive overjet or posterior crossbite have been related to TMD. Furthermore, orthodontic treatment as a contributing factor for the development of TMD has been the subject of many studies, (4) especially after the Michigan Court in 1987, when an orthodontist was damned to pay a $850,000 compensation to a patient as he was considered main responsible of the TMD developed after the orthodontic treatment (5). Nevertheless, this topic still remains under discussion. Arguments against the orthodontic treatment are usually based on the deleterious effects on stomatognathic function such as occlusal interferences, consequences of the use of intermaxillary elastics, extraoral forces or functional appliances. On the other hand, several studies demonstrate no relation between orthodontics and TMD (6).

Signs and symptoms of TMD are relatively common on adolescents as several longitudinal studies have shown that clinical signs of TMD increase with age, appearing especially during the second decade of life (7,8). However, they are inconsistent over the course of time, showing both improvement and impairment on an individual basis.

Moreover, approximately 30% of western European children and adolescents seek orthodontic treatment (9), thus the consideration of orthodontics as a risk factor for the development of TMD may stand in a time-related coincidence. Even though, some evidence has been presented against orthodontics, the relationship between TMD and orthodontic treatment is still unclear. The cause-effect relationship between TMD and orthodontic treatment is difficult to demonstrate because of the incidence of TMD among people of an early age (7) and therefore they could show signs and symptoms of TMD either before, during or after ort-hodontic treatment.

The aim of this systematic literature review was to answer the following question: Is there any association between the signs and symptoms of TMD and orthodontic treatment?.

## Material and Methods

-Search Criteria 

An electronic research was conducted in PubMed-Medline databases covering the period from January 2000 to August 2013 using as keywords “orthodontics and temporomandibular disorders”, “orthodontics and facial pain”, “malocclusion and temporomandibular disorders”, “orthodontics and temporomandibular disorders treatment”. Studies associating sleep apnea, craniofacial syndromes, and treatment with orthopedics or orthognathic surgery were not included as well as those that reported only the association between malocclusion and TMD. The search strategy was performed by two calibrated reviewers (FJ.F.G. and A.C.O.), independently applied the inclusion and exclusion criteria to every article, with an adequate concordance being shown (kappa index, 0.86). Disagreements between the 2 reviewers were discussed with a third reviewer (JL.L.C) for consensus. Articles wherein at least one of the reviewers felt that reflected the purpose of this study were reviewed in their entirety. Selected article references were reviewed in order to extend the search for relevant articles. The evidence grade of the included studies was judged to be strong, moderately strong, or limited. The included papers were evaluated by all members in order to ensure they match the inclusion criteria. Studies assessing orthodontic treatment of TMD, also those concerning orthodontics and TMD were included, manual searches of journals and books concerning orthodontic treatment and TMD also were reviewed. Only human studies in English and French languages were considered.

-TMD Diagnostic Criteria

Only, those studies in which the diagnosis of TMD included significant problems in the temporomandibular joints or muscles verified by clinical examination were accepted. Studies relating signs and symptoms of TMD and different types of malocclusions treated with orthodontics were also included. Studies based on the use just of a diagnostic index and without clinical examination were not considered.

The following criteria for inclusion or exclusion were used:

-Criteria for Inclusion

• Studies considering orthodontics as a risk factor on the development of TMD 

• Only prospective, longitudinal, case-control or retrospective studies with a large sample (n>100 patients) and significant statistical analysis were included

• English or French language 

• Studies were qualified with strong evidence (A) or Moderately strong evidence (B) according to the study quality from the Centre for Reviews and Disseminations in York, United Kingdom (10):

▪Strong evidence (A)

Randomized controlled trial, prospective studies/large study samples

Well-defined and adequate control group

Clearly defined and clinically relevant variables

Low dropout rate

Relevant statistical analysis

▪Moderately strong evidence (B)

Prospective study, cohort, controlled clinical trial, or well-defined retrospective study with large study group

Clearly defined and clinically relevant variables

Low dropout rate 

Relevant statistical analysis

▪Limited evidence (C)

Cross-sectional study

Clinically inadequate result variables

High dropout rate

No control group of its own in the study 

Limited/no statistical analysis

Addressing the issue in question only in part 

## Results

Search and Quality Assessment Results

The search strategy resulted in 61 articles. Forty-seven papers were excluded because they did not fulfil the inclusion criteria. Five papers (11-15) were in according with the scope of this review but according to our inclusion criteria with limited evidence and are listed in  Table 1. After selection, nine articles (6,16-27) qualified for the final analysis.  Table 2 shows and summarizes the most recent articles that link orthodontics with TMD published between 2000 and 2013. They were divided in groups by author, type of study, purpose, population, conclusions and grade of evidence ( Table 2,Table 2 Cont).

**Table 1:**
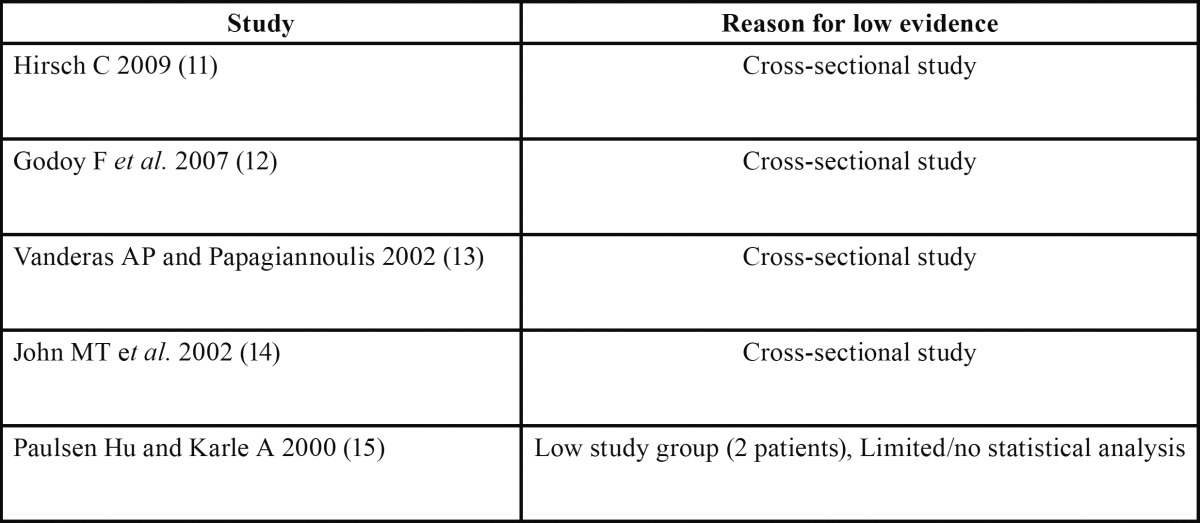
Low Evidence Studies.

**Table 2:**
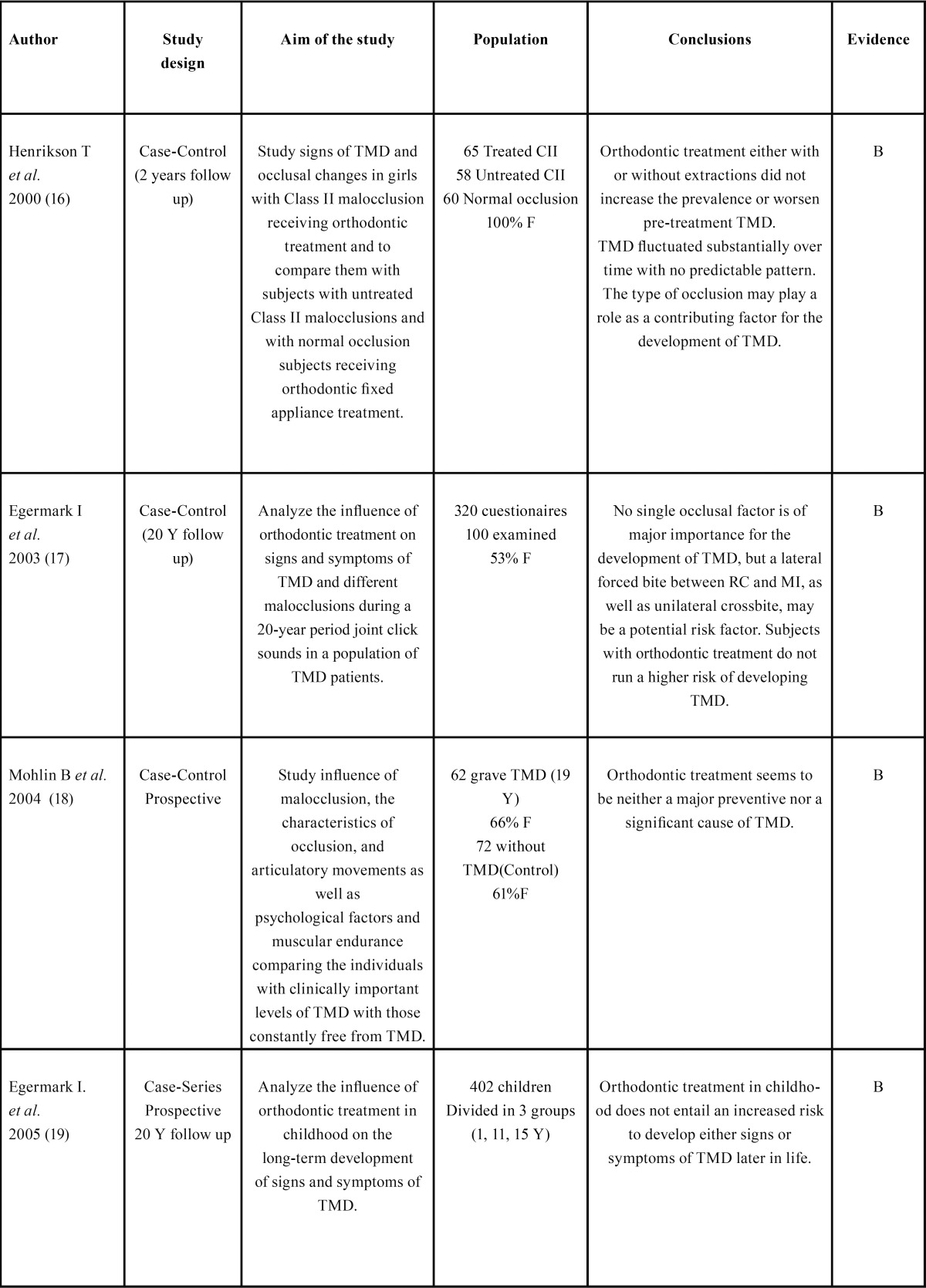
Quality studies that related orthodontics and TMDa.

**Table 2 Cont:**
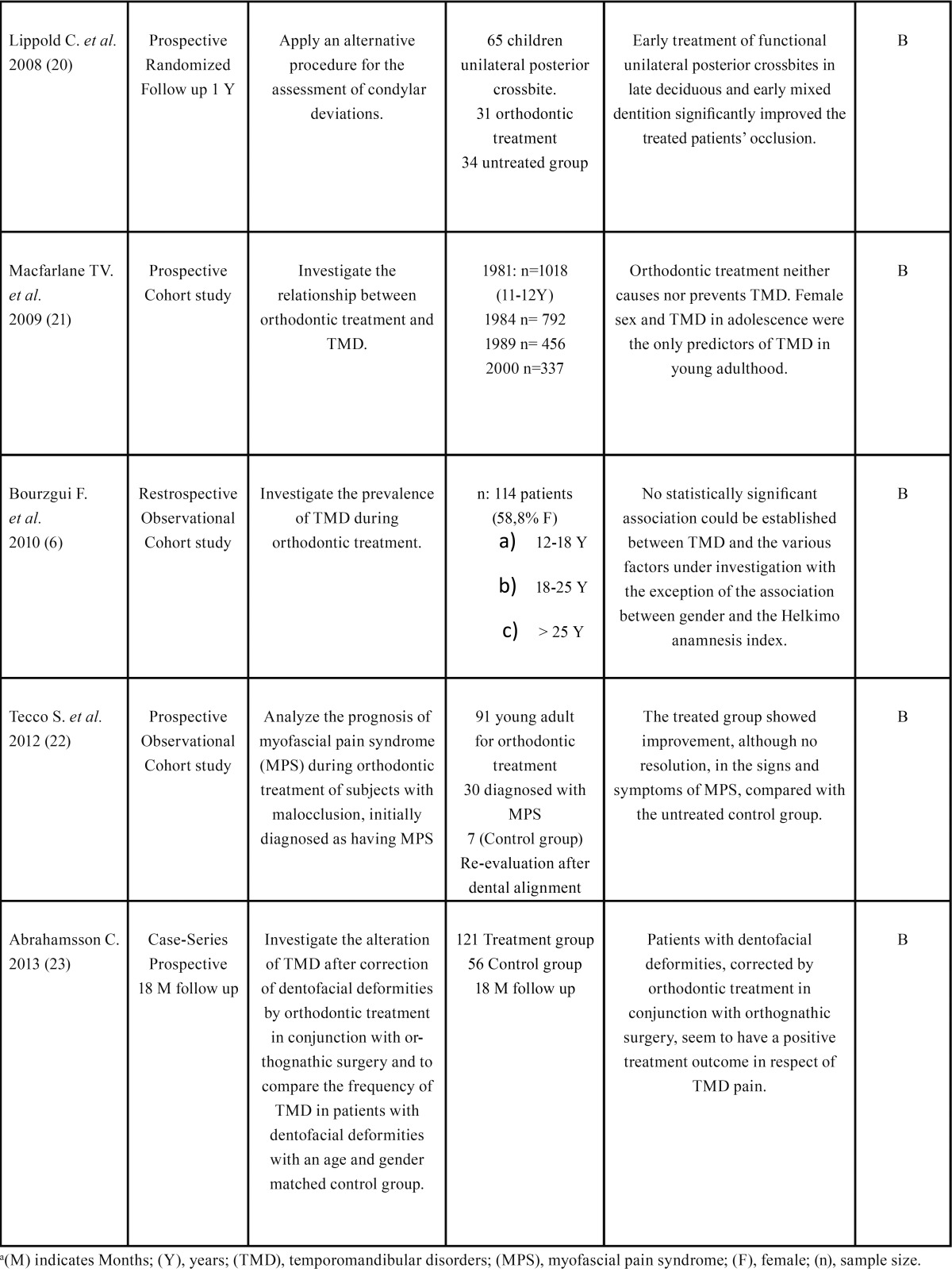
Quality studies that related orthodontics and TMDa.

None of the selected articles found a deleterious effect of the orthodontic treatment on the temporomandibular joint. Two of them found that orthodontic treatment could reduce signs and symptoms of TMD (21,22). The majority of them showed a relationship between TMD and female sex and a fluctuation of its manifestations over time. The differences in TMD between those with and without malocclusion were small. Subjects with untreated crossbite, crowding or large overjet showed a higher prevalence of signs and symptoms of TMD (15,16)

## Discussion

The possible relationship between orthodontics and TMD commands great interest in the contemporary literature. Nevertheless, despite the volume published, the mechanism whereby orthodontic treatment might influence the aetiology of TMDs is still unknown.

Here we analyse the role that orthodontics might play in the initiation of TMDs. The assessment and analysis of the numerous papers published concerning the negative effects of orthodontics on the stomatognathic system become difficult due to the heterogeneity of the variables and the methodology used to record results. Although 75% of the population may show clinical signs of TMD (19).

The diagnostic criteria defining this pathology until now have not been standardized. Recently, in 2014 the international RCD/TMD Consortium Network and orofacial pain special interest group has published their recommended evidence-based new diagnostic criteria for temporomandibular disorders for clinical and research applications. This protocol has been shown appro-priate for use in both clinical and research setting, being considered as a validated instrument for identification of patients with a range of simple to complex TMD presentations. Nevertheless this protocol is still not frequently used in the current practice nor in the research sphere. Thus, the assessment and comparison of the different publications become difficult.

It should therefore be considered that orthodontists without appropriate training might not take into account the function of the temporomandibular joint and the risk for TMD could be increased (24). On the other hand, the multifactorial character of TMDs (occlusion, trauma, emotional stress, severe pain and parafunctional activity) (8,13) and their great diversity of manifestations make difficult to prove that orthodontics will solve or improve a TMD.

Longitudinal studies (17,21) have shown an increase in the prevalence of signs and symptoms of TMD with age, with a greater prevalence of signs than symptoms, therefore it is important to include a comprehensive physical examination as part of the diagnostic process, regardless the type of orthodontic treatment to be performed. Machen *et al.* (25,26) in their studies of 1990 and 1991 already emphasized the need to record any alteration diagnosed on clinical examination of the temporomandibular joint (TMJ) for medico-legal reasons. They also recommended the control of the TMJ situation every 6 months during the orthodontic treatment and the sign of an informed consent by the patient.

Since Costen (27) first associated occlusal factors with TMD symptoms in the 1930s, different types of treatment have been proposed, including orthodontics and occlusal adjustments to correct malocclusions and improve signs and symptoms of TMDs. Achieving an ideal occlusion through orthodontic treatment and/or occlusal adjustments might decrease signs and symptoms of TMD.

In the case of sagittal malocclusions, several studies consider it a contributing factor for the development or perpetuation of TMD. Henrikson and Nilner (9), in a study conducted on 183 patients, reported lower prevalence of signs and symptoms of TMD in patients with class I malocclusion than in those with class II, although this influence was difficult to quantify and predict. They also find significantly fewer functional occlusal interferences in the class II group treated with orthodontics than in the group with untreated malocclusion and the group with normal occlusion, which could explain the decreased muscular signs observed in this group of patients. These results emphasize the importance of a correct and stable occlusion for the proper functioning of the stomatognathic system.

Additionally, Egemark *et al.* (19) analysed the influence of multiple variables on TMDs in three samples of children of 7, 11 and 15 years, reporting morphological criteria such as class II, class III, anterior open bites and posterior crossbites as potential factors of predisposition to TMDs associated with functional malocclusions. Moreover, in a previous research, they described an impro-vement in muscular signs after orthodontics in class II malocclusions (17), which could be explained by the improved occlusal stability observed by reduction of interferences and increase in occlusal contacts in treated patients. This improved muscular discomfort may already be noted during orthodontic treatment, probably owing to the diminished activity of the chewing muscles during treatment brought on by the increased dental sensitivity associated with orthodontic movement. Likewise, Vanderas and Papagiannoulis (13), in their multiple logistic regression study, analysed a sample of 314 children aged 6-8 assessing clinical signs of TMDs and also morphological or functional malocclusions. Prognathism was basically associated with TMJ noises, whereas posterior crossbites had a significant impact on joint pain. They concluded that parafunctional habits and certain structural and physiological factors may increase the probability of developing signs and symptoms of a TMD in children. Other studies, however, could not demonstrate a correlation between prognathism and TMD (11,28).

Among different malocclusions, posterior crossbite are considered to have a strong impact on the functioning of the stomatognatic system. Several studies have associated unilateral posterior crossbite in children with an increased probability of developing signs and symptoms of TMD (12,29). The mandibular deviation that is frequently associated with this posterior crossbites, interferes with the development and growth of the stomatognathic system (30). Lippold *et al.* (20) studied the discrepancies in the condyle position between the centric relation and maximum intercuspation in a sample of 65 children with posterior crossbite in mixed dentition. A comparison of patients who had received orthodontic treatment and others who had not, revealed no statistically significant differences between the groups at the beginning of treatment, being the condyle deviation less than 2mm in the transverse, frontal and sagittal planes on both sides. The treated group showed a statistically significant decreased in condyle deviation.

Regarding the possible consideration of orthodontic treatment as a TMD risk factor, several authors (4,12) consider that certain dental interventions, including orthodontics itself could cause TMD. However, the prospective cohort study by MacFarlane and co-workers29 concluded, after a follow-up period of 20 years, that orthodontics is not linked with the appearance of TMDs or their persistence. Only female gender and the presence of signs and symptoms of TMD during adolescence were the unique pre-dicting factors. The logistic regression analysis showed an odds ratio of 3.0 and a confidence index of 95% between 1.2 and 8.2 for the female gender and an odds ratio of 4.5 between 2.0 and 10.0 for the TMD in adolescence.

It is necessary to take into account the fluctuating nature of TMDs, which could be wrongly attributed to be caused or aggravated by orthodontics due to the fact that this is the period of the life when this treatment is usually carried out (16). Epidemiological studies like Magnusson *et al.* (31) revealed a high prevalence of signs and symptoms of TMD, especially TM joint noises, in children and young people, with the greatest prevalence in those aged between (15-25). Due to this fluctuating and unpredictable behaviour of the TMD, it results of utmost importance to properly inform the patients about the high prevalence of this condition and its multifactorial nature, which makes difficult to establish an association with the orthodontic treatment performed. Therefore, the continuous monitoring of TMJ is essential to detect the onset of a TMD as early as possible. In these cases it is recommended to temporarily stop orthodontic treatment in order to avoid possible aggravating factors until signs and symptoms, especially pain, improve. Otherwise, if TMD is diagnosed in the first evaluation of the patient, the orthodontic treatment should not be initiated, according to Michelotti *et al.* (32) as long as the patient suffers from a facial pain. In both cases, the priority should be the differential diagnosis between musculoskeletal condition and another diseases, and the management of the TMD would include the use of occlusal splints to evaluate the interference-free position of the mandible, pharmacotherapy, behavioural therapy, and/or physical therapy.

## Conclusions

After a detailed analysis of the studies found in the current literature, we concluded that:

a) Associations between specific types of malocclusions and development of significant signs and symptoms of TMD could not be verified.

b) According to the authors studied, there would appear to be no evidence for a direct or obvious cause-effect relationship between orthodontic treatment and TMD.

c) The differentiation of patients into control and study groups in the studies design is a persistent issue.

d) Different therapeutic methods are used to treat TMD with orthodontics. Therefore, there is still a need for longitudinal and randomized trials.
